# Supplementary material for: Enhanced Microwave Absorption Bandwidth in Graphene-Encapsulated Iron Nanoparticles with Core–Shell Structure
Source: Nanomaterials (Basel). 2020 May 12;10(5):931. doi: 10.3390/nano10050931 (PMC7279258; doi:10.3390/nano10050931)
Supplement: Supplementary file 1 [file nanomaterials-10-00931-s001.pdf]

nanomaterials

Supporting information

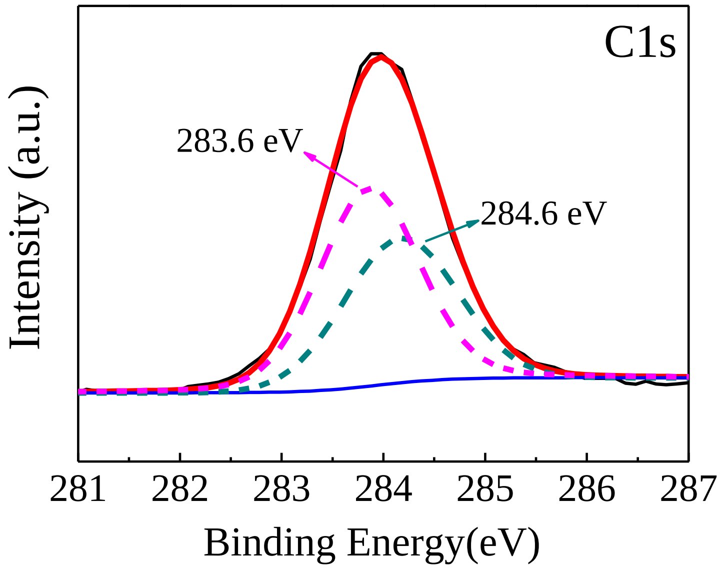

Figure S1. C1s XPS spectra of the Fe(G) nanoparticles.

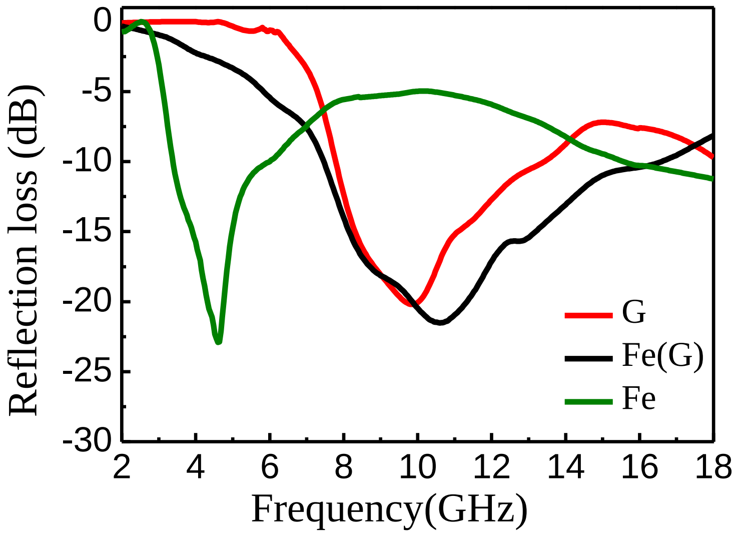

Figure S2. Frequency dependences of R for a Fe(G)/paraffin composite with 40 wt% loading as compared to only graphene (G) and only iron nanoparticles (Fe) of 3 mm in thickness under 2–18 GHz.
